# IMPROVING STUDENT CONFIDENCE IN APPLYING GERIATRICS BEST PRACTICES USING THE 4MS FRAMEWORK

**DOI:** 10.1093/geroni/igae098.3857

**Published:** 2024-12-31

**Authors:** Jennifer DeGennaro, Leonard Powell, Christian White, Kevin Overbeck

**Affiliations:** Rowan-Virtua School of Osteopathic Medicine, Stratford, New Jersey, United States; Rowan University, Stratford, New Jersey, United States; Rowan University, Stratford, New Jersey, United States; Rowan University, Stratford, New Jersey, United States

## Abstract

Recognized by the Institute for Healthcare Improvement as a Level II Age-Friendly Health System, the Rowan-Virtua School of Osteopathic Medicine (RVSOM) Department of Geriatrics and Gerontology is committed to infusing the 4Ms framework (What Matters, Medication, Mentation, Mobility) into all education initiatives as well as clinical practice. RVSOM medical students are exposed to the 4Ms in the second year of undergraduate courses and the framework is further emphasized during the required 4-week clerkship. To standardize content delivered to students rotating at a host of rotation sites and to augment the assigned self-study modules, a new initiative was introduced at the beginning of academic year (AY) 2024-2025. Students now participate in a full day of in-person didactics designed to teach them to use evidence-based screening tools and employ best practices when caring for older patients. A post-clerkship survey on the 4Ms approach was administered in AY 2023-2024 which allows an examination of differences between cohorts of students who received the in-person didactics and those who did not. Analysis of the data indicates that students benefit from the focused didactics as significant differences emerged on nearly half of the survey items [e.g., sufficient knowledge to perform a Geriatric Assessment [t(109) = 2.45, p =.016, d =.47] and confidence in initiating a conversation about Advance Care Planning [t(109) = -2.69, p =.008, d = -.51]). Findings indicate that enhanced training and exposure to Geriatrics best practices bolsters students’ confidence in understanding geriatrics issues and assessing older patients.

